# Evaluating the Sustainability of High-Dose Sewage Sludge Application in Fertilizing Szarvasi-1 Energy Grass Plantations

**DOI:** 10.3390/plants15030392

**Published:** 2026-01-27

**Authors:** Ferenc Fodor, Péter Nyitrai, Éva Sárvári, Csaba Gyuricza, Gyula Sipos

**Affiliations:** 1 Department of Plant Physiology and Molecular Plant Biology, ELTE Eötvös Loránd University, Pázmány Péter Lane 1/c, 1117 Budapest, Hungary; peter.nyitrai@ttk.elte.hu (P.N.); eva.sarvari@ttk.elte.hu (É.S.); 2 Institute of Agronomy, Hungarian University of Agriculture and Life Sciences, Páter Károly Street 1, 2100 Gödöllő, Hungary; gyuricza.csaba@uni-mate.hu; 3 Agricultural Research and Development Institute, Szabadság Street 30, 5540 Szarvas, Hungary; gyula.sipos@sonic.co.hu

**Keywords:** biomass plant, element composition, *Elymus elongatus*, phytoremediation, *Thinopyrum obtusiflorum*

## Abstract

The accumulation of municipal sewage sludge is a worldwide problem, although when properly treated, it can be utilized for various purposes in industry and agriculture. Due to its high nutrient content, one of its possible uses is the application as fertilizer on agricultural or degraded lands with the purpose of non-food plant production. In the present study, the sustainability of dehydrated sewage sludge application was tested in Szarvasi-1 energy grass (*Thinopyrum obtusiflorum* cv. Szarvasi-1) plantations, with special focus on the turnover of nutrients and trace elements in two experiments conducted outdoors between 2016 and 2019. Experiment 1 was conducted in 1 m^3^ containers, and the treatment was started on two-year old plants in 0, 15, 22.5, and 30 Mg ha^−1^ doses per year applied in two or three portions to reveal the upper limit of sludge application. Experiment 2 was conducted in 100 m^2^ field quadrates with 0, 7.5, 15, and 22.5 Mg ha^−1^ doses per year applied once a year, which is in the range of the currently permitted application dose in Hungary. Soil, sludge, and plant samples, as well as physiological data, were collected. Aboveground biomass yield was measured 2–3 times per year. Increasing doses of sewage sludge significantly increased the yield compared to the controls, but the increment between the second and third doses was small. Chlorophyll content (SPAD values) increased tendentiously and partly significantly. The maximal quantum efficiency of PSII and the stomatal conductance was not improved compared to the control, whereas the relative water content of the plants was increased in Experiment 1 but not in Experiment 2 compared to the control. Malondialdehyde concentration was increased by the largest dose in Experiment 1. The concentration of macroelements, Ca, Mg, N, and S, increased in the aboveground biomass with increasing doses of sewage sludge, but even after three years, the cumulative amount removed with the harvested biomass was much smaller than the amount remaining in the soil. The total amount of K in the harvested biomass exceeded that introduced to the soil by the treatments. Micro- and trace-element concentrations did not show increasing tendency in the biomass, suggesting a slower uptake and removal rate than macroelements. The results point to the necessity to assess the real nutrient requirement and trace-element uptake by the plants as compared to the sewage sludge treatment to avoid their uncontrolled accumulation in the soil and ensure a sustainable fertilization of the plantations.

## 1. Introduction

One of the major environmental problems at global scale is the accumulation of sewage sludge, the byproduct of urbanization. Wastewater treatment plants produce and settle tons of sewage sludge that are treated and disinfected but still contain pollutants as well as nutrient elements in various concentrations. The methods of decontaminating and utilizing sewage sludge can vary, but its quality fundamentally influences the options for its subsequent use and disposal [1,2,3,4]. Sewage sludge management includes reclamation and recultivation of land; plant cultivation not intended for consumption or for production of food; usage in agriculture; usage in architecture; recovery of phosphorus, rare earth metals, or fats and usage in industry; producing combustible pellets and granulates; additive for biogas production; and storage on territory of the treatment plants and landfills [5,6]. In Hungary, the most significant of these is agricultural placement, the method and conditions of which are regulated by Government Decree 50/2001 in accordance with European legislation [7].

Sewage sludges—in addition to their high macronutrient content (N, P)—generally also contain micronutrients in high (and in some cases toxic) concentrations (e.g., Cu, Zn, Ni). Thus, it is not surprising that in the European Union sewage sludge has long been used, and in increasing amounts, in agriculture for soil improvement and nutrient replenishment [6]. On the other hand, toxic elements (e.g., Cd, Pb, Cr), organic pollutants (e.g., polycyclic aromatic hydrocarbons, etc.), pathogenic microorganisms, and microplastics may also remain in sewage sludge, depending on the treatment [8]. A portion of all the toxic substances introduced into the soil may become bound, chemically or biologically degraded or transformed, while another portion may be taken up by plants [9,10,11]. The amount of metal that can be taken up depends on the plant species and variety, the pH of the sludge and soil, as well as the moisture content, organic matter content, and cation exchange capacity of the soil [12,13].

Degraded or even contaminated lands are not suitable for agricultural production of consumable plant products but may undergo phytoremediation or just be used for the production of large biomass crops for industrial purposes such as bioenergy production [13]. Quickly growing plants require frequent resupply of nutrients to the soil, for which sewage sludge is a cost-effective alternative to conventional fertilizers [9].

Significant knowledge is already available on the growth, physiological characteristics, and partly the metal-tolerance and metal-accumulation capacity of the Szarvasi-1 energy grass (*Thinopyrum obtusiflorum* cv. Szarvasi-1; syn. *Elymus elongatus* subsp. *ponticus*, *Agropyron elongatum*, *Elytrigia elongata* (*Poaceae*, *Triticeae*)), a high biomass plant developed for industrial purposes [14,15]. It tolerates salinity (NaCl), Zn, and Pb well, but is sensitive to Cd; however, S-methylmethionine treatment can enhance its Cd tolerance [16,17]. In the year following sowing, its biomass yield is already substantial, and this production may reach its maximum from the second year onward. As a perennial grass species, its plantation can be maintained for up to 15 years without reseeding. Both its shoot and root can reach a length of two meters [14]. Based on all these characteristics, it may be suitable for the decontamination and utilization of sewage sludge, as well as for biomass production for bioenergetic purposes in areas treated with sewage sludge.

In the present study, based on the previous knowledge, increasing doses of dehydrated municipal sewage sludge were applied in two experiments as substitute to conventional fertilizers in order to supply the plants with sufficient amount of nutrients for maximizing the yield. In Experiment 1, large aluminum containers were used to grow the plants, and treatments were started when the plants were two years old, whereas, in Experiment 2, new plantations were established on 100 m^2^ quadrates, both in field conditions. The main goals of the study were to reveal the effects of high sewage sludge doses on the physiological characteristics and yield of the plants, as well as the sustainability of such treatments in terms of element uptake, accumulation, and nutrient balance between the amounts deposited in sludge and those harvested in plant biomass.

## 2. Results

### 2.1. Dry Biomass Yield of the Szarvasi-1 Energy Grass

**Figure 1:**
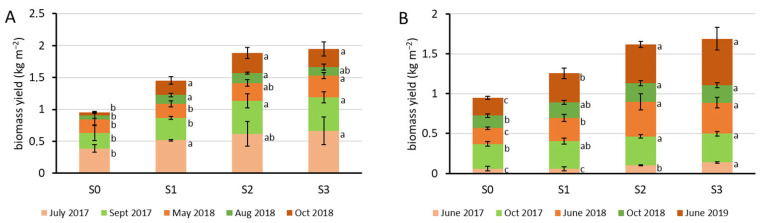
Total aboveground biomass dry matter yield of Szarvasi-1 energy grass grown in containers (Experiment 1, (**A**)) and field quadrates (Experiment 2, (**B**)) harvested between 2017 and 2019 in one square meter soil surface. Stacked columns represent the cumulative data per treatment with increasing doses of sewage sludge (mean ± SD, n = 3). Significant differences at *p* < 0.05 are indicated between the treatments at each harvest time with different lowercase letters (one-way ANOVA with Tukey’s HSD post hoc test).

In Experiment 1, the plants were three years old when the first yield assessment was performed. As the plants were irrigated, water supply did not limit their growth, making it possible to harvest twice or three times per year. Total aboveground biomass dry matter yield of the containers treated with increasing sewage sludge doses (S1, S2, S3) significantly exceeded that of the untreated control ones (S0) already in the first year (Figure 1A). In the following harvest occasions, there was a slight variability concerning the significance of yield increment compared to the control, but considering the two-year cumulative result by October 2018, the yield was almost double in S2 containers compared to S0, which was hardly exceeded by S3 containers. In Experiment 2, the first harvest was conducted less than one year after sowing, whereas the plantation was two and a half years old at the last harvest—hence the great difference in yields (Figure 1B). The yield of the S1 treatment significantly exceeded that of S0 first in the second year, whereas S2 and S3 treatment did so at the first harvest. Cumulative yield increased with increasing sewage sludge doses S1 and S2, but for S3 the increment was not considerable, similar to Experiment 1.

### 2.2. Physiological Characterization of the Plants Under Sewage Sludge Treatments

**Figure 2:**
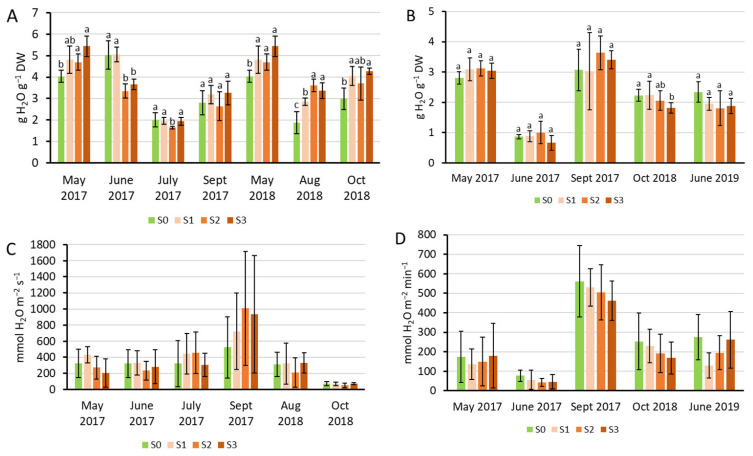
Relative water content in the total aboveground biomass (calculated for 1 g dry weight, DW) (**A**,**B**) and stomatal conductance (**C**,**D**) of the youngest fully developed leaves of Szarvasi-1 energy grass grown in containers (Experiment 1, (**A**,**C**)) and field quadrates (Experiment 2, (**B**,**D**)). Columns represent the mean ± SD, n = 3 for relative water content and n = 9 for stomatal conductance. Significant differences at *p* < 0.05 are indicated between the treatments at each harvest time with different lowercase letters (one-way ANOVA with Tukey’s HSD post hoc test). In (**C**,**D**), no significant difference was found at *p* < 0.05 among the treatments.

The significant growth stimulation observed in both experiments was coupled with physiological stability of the Szarvasi-1 plants. In Experiment 1, the relative water content of the plants increased compared to the control in May 2017 and all sampling occasions in 2018 (Figure 2A). However, it decreased in June and July 2017. In Experiment 2, the relative water content did not change except for a slight decrease in October 2018 (Figure 2B). In general, the water content was larger in the spring growth period and smaller in summer, except for September 2018 in Experiment 2, when rainy days preceded the sampling. The stomatal conductance did not show variability due to treatments but varied among measurement times due to different field conditions in the two Experiments (Figure 2C,D).

**Figure 3:**
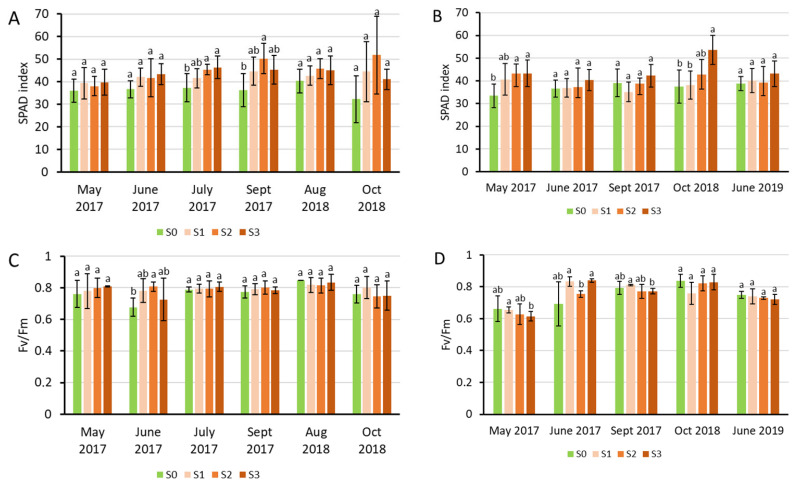
Relative chlorophyll content (SPAD index) (**A**,**B**) and the maximal quantum efficiency of the PSII (Fv/Fm) (**C**,**D**) measured in the youngest fully developed leaves of Szarvasi-1 energy grass grown in containers (Experiment 1, (**A**,**C**)) and field quadrates (Experiment 2, (**B**,**D**)). Columns represent the mean ± SD, n = 3. Significant differences at *p* < 0.05 are indicated between the treatments at each harvest time with different lowercase letters (one-way ANOVA with Tukey’s HSD post hoc test).

The SPAD values representing the chlorophyll content showed tendentious increases upon sewage sludge treatments in the youngest fully developed leaves of Szarvasi-1, but the increment compared to the untreated control became significant only in July and September 2017 in Experiment 1, and in May 2017 and October 2018 in Experiment 2 (Figure 3A,B).

The maximal quantum efficiency of the photosystem II (PSII, Fv/Fm) was in most cases at the optimal value, except in June 2017 (Experiment 1), when the control values were significantly lower, and in May 2017 (Experiment 2), when all measured values (S0–S3) were between 0.6 and 0.7, referring to stress in the young plants (eight months after sowing). In this latter case, the treatment tendentiously decreased the Fv/Fm, but only the S3 value became significantly lower than the control S0.

**Figure 4:**
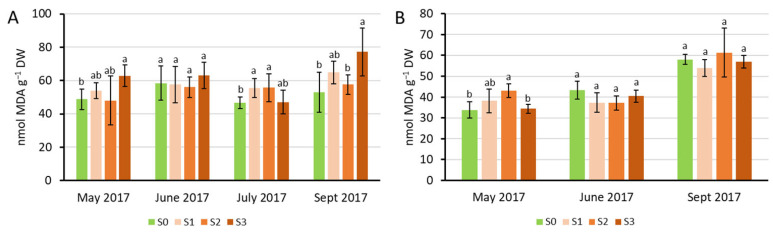
Malondiadehyde concentration measured in the youngest fully developed leaves of Szarvasi-1 energy grass grown in containers (Experiment 1, (**A**)) and field quadrates (Experiment 2, (**B**)). Columns represent the mean ± SD, n = 3. Significant differences at *p* < 0.05 are indicated between the treatments at each harvest time with different lowercase letters (one-way ANOVA with Tukey’s HSD post hoc test).

The concentration of malondialdehyde (MDA), a byproduct of lipid peroxidation, remained at control level in the youngest fully developed leaves in Experiment 1 in June 2017, while S3 treatment in May and September 2017 and S1 and S2 in July increased the MDA levels. In Experiment 2, a significantly increased MDA level was only detected in S2 treatment in May 2017 (Figure 4A,B) (MDA was not measured in 2018 and 2019).

### 2.3. Changes in the Element Composition of the Plants Under Sewage Sludge Treatments

The element composition of the plant material was expected to be modified by the sewage sludge treatments. The high nutrient content of the dehydrated (centrifuged) sewage sludge product of the Budapest Sewerage Works PLC is coupled with a very low concentration of nonessential heavy metals or trace elements that do not exceed the allowance limit of the 50/2001 Government Decree, which regulates the application of sewage sludge and sewage sludge compost in agricultural utilization, both on the basis of maximum applicable yearly amount and maximum contaminant concentrations (Table 1) [7]. So, the risk of heavy metal accumulation in the soil and in the plants was low.

**Figure 5:**
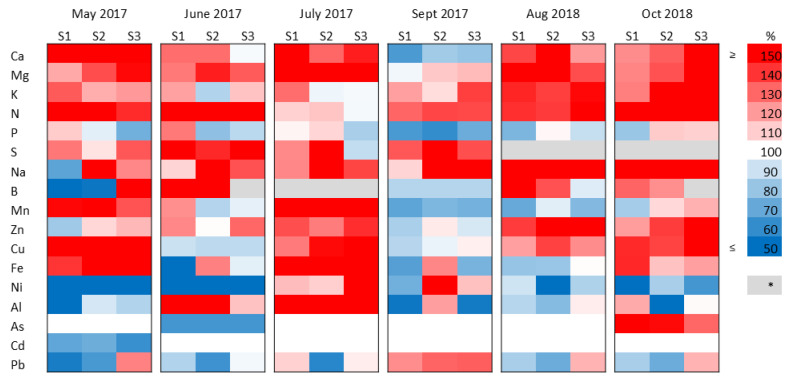
Heat map showing changes in element concentrations in the aboveground biomass of Szarvasi-1 energy grass treated with increasing doses of sewage sludge (S1–3) as the percentage of the untreated control, sampled at each harvest occasions in Experiment 1. Each colored panel represents the results of three pooled samples. * Missing data. 100% represents no change compared to the control.

The aboveground plant material has been sampled to determine the element concentrations at each harvest time. In Experiment 1, most macroelement concentrations (Ca, Mg, K, N, S) increased considerably after the treatments compared to the control samples, except for Ca in September 2017, which decreased, and P, in all treatments, which fluctuated between slight increase and decrease (Figure 5). Na also showed a generally increasing trend. Microelements and trace elements (B, Mn, Zn, Cu, Fe, Ni, Al, As, Cd, Pb) showed considerable variability in response to treatments. Zn and Cu generally increased, and Ni generally decreased, whereas B, Mn, Fe, Al, and Pb varied among sampling times. The decreases and increases in As and Cd concentrations can be considered miniscule, as these trace elements were also present in the plants and in the soil in very low amounts (Table 2 and Table S1). In July 2017, a summer harvest sampling showed increased element concentrations for almost all treatments, whereas the following September showed an opposite trend, except for Mg, K, N, S, and Na.

**Figure 6:**
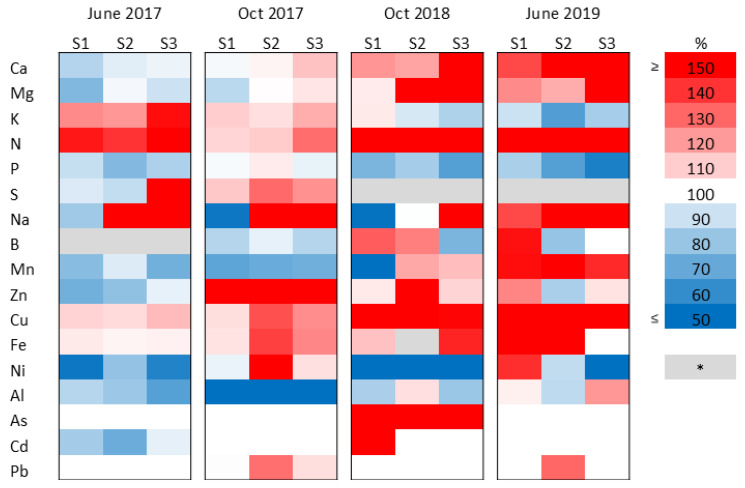
Heat map showing the changes in element concentrations in the aboveground biomass of Szarvasi-1 energy grass treated with increasing doses of sewage sludge (S1–3) as percent of the untreated control, sampled at each harvest occasions in Experiment 2. Each colored panel represents the results of three pooled samples. * Missing data. 100% represents no change compared to the control.

In Experiment 2, the Ca and Mg concentrations first decreased compared to the control, then started to increase in later harvest occasions, and this trend was generally observable with increasing sewage sludge doses (Figure 6). Potassium concentration showed the opposite change, while Na generally decreased in low-dose treatments (S1) and increased considerably in high-dose treatments (S2, S3). Nitrogen considerably increased in the plants, except for October 2017, when it showed only a moderate increase, whereas P changed oppositely. S and B concentrations showed varying changes, and due to some missing data, the effect of the treatments on these elements cannot be observed reliably. The microelements Mn, Zn, Cu, and Fe first showed slight decreases or increases compared to the control, then, in later harvest times, they generally turned to increased level in the plants. This change is more marked in case of Zn and Cu. As opposed, Ni and Al showed decreased levels compared to the control throughout the experiment, except for October 2017, when Ni increased with increasing doses. The trace elements As, Pb, and Cd showed very small fluctuations similar to Experiment 1.

When the concentration of each element was averaged for all the sampling occasions to see overall changes throughout the experiments, we found no significant changes due to considerable seasonal variation, but indeed increasing trends with sewage sludge doses could be identified (Table 2). In Experiment 1, Ca, Mg, K, N, S, Na, Zn, Cu, and Fe well represented this observation, while changes in the P, B, Mn, Ni, and Al concentrations were not directly connected to increasing sewage sludge doses. In Experiment 2, very similar changes were noted: Ca, Mg, N, S, Na, Zn, and Cu showed increase, while P decreased with increasing treatment doses (Table 3). In the case of K, B, Mn, Fe, Ni, and Al varying concentrations were observed.

### 2.4. Nutrient and Trace-Element Balance

We have performed a lot of measurements to analyze the nutrient and trace-element changes in the soil, but the obtained data were not informative and did not show clear changes after the sewage sludge treatments (Tables S1 and S2). For this reason, we chose to compare the total amount of elements introduced with the treatments and removed by harvesting the aboveground biomass of Szarvasi-1 and calculated the cumulative element use efficiency (CEUE) (Table 4 and Table 5). (The missing data for the elements at some harvest times were completed using the average of the other measurement dates with reference to Figure 5 and Figure 6 and Table 2 and Table 3). In general, the amounts of elements introduced to the containers and quadrates greatly exceeds the amount removed by harvesting the biomass: the ratio remained at about 0.1 or lower. This did not change when one-year or two-year data were calculated in Experiment 1 probably because the treatments were repeated in consecutive years. In Experiment 2, the CEUE increased when one, two, or three years were included in the calculation. (In this experiment, there was no treatment in the third year).

Based on these data, the hypothetical total removal of all the introduced elements would require 10–20 years for macroelements and 20–100 years for microelements, unless further treatment is made. However, there are exceptions, such as K among the macroelements, for which the removal efficiency exceeded the amount introduced with treatments by 2.3–7.3 times. It must be noted that As and Cd are taken up more efficiently than the essential microelements, except B. It should also be noted that the calculation assumes stable long-term yield, similar element concentrations, and climate conditions, as well as the same scheme of plantation management.

When the increasing sewage sludge doses are considered, the CEUE decreased in the order S1 > S2 > S3 in most cases. However, in Experiment 1, S, Na, and Fe removal increased in S2 treatment then decreased in S3 compared to S1. Ni removal increased in S3 only. In Experiment 2, Na and Mn removal increased in S2 and decreased in the S3 treatment, whereas there was no considerable change in Fe.

## 3. Discussion

Municipal sewage sludge has been proved to be a valuable fertilizer material when properly pretreated for agricultural application. Furthermore, it can be applied to improve the characteristics of degraded or contaminated soil [18,19,20]. The amount to be applied to (agricultural) land is regulated in the European Union and at the national level [21,22]. However, sludge is produced at such a rate that it accumulates and should be disposed of at least in part, whereas the production of biomass plants for industrial purposes (i.e., energy plants) needs low-cost cultivation techniques in the long term. So, in the present study, the sewage sludge product was applied in experimental Szarvasi-1 energy grass plantations to assess the feasibility of its sustainable recycling.

### 3.1. Yield and Physiological Characteristics

In both experiments, the sewage sludge application resulted in significant increases in yield already at the first harvest after treatment, but this growth stimulation became continuous from the first to the last harvest only at the highest dose, S3. The yield changed alternately, being higher in the spring–summer period and lower in the autumn period. This is because the flowering stems are developed usually in the former, constituting a much larger biomass compared to the leafy vegetative growth in the latter. The cumulative yield in the S3 treatment reached a 100% increase in Experiment 1 and almost 80% in Experiment 2. In previous studies, there are similar observations concerning the yield increasing effect of sewage sludge [8,9,10,23]. However, as increasing the treatment dose from S2 to S3 did not result in further significant yield increment, the biomass gain alone does not justify more than 22.25 Mg d.m. ha^−1^ year^−1^ in older plantations and more than 15 Mg d.m. ha^−1^ year^−1^ in newly established plantations, which is in good agreement with a six-year-long field study conducted on cup plants (*Silphium perfoliatum* L.) [24]. Nevertheless, there are other benefits of applying sewage sludge to improve the properties of poor soil, e.g., organic matter content [19,25].

The nutrients in the applied treatments had slightly positive or no effect causing physiological change in the plants compared to the control. The relative water content increased in both experiments at several sampling times, while it decreased only in a few samples. This would imply that higher nutrient availability and accumulation, especially N, K, and Na possibly contributing to decreases in tissue water potential, resulted in more efficient water uptake and preservation [23,26]. Stomatal conductance did not change significantly in response to treatment.

The SPAD index, referring to the total chlorophyll content of the leaves, showed a clear stimulating trend, becoming significant at four measurement occasions in the two experiments. The peak values were measured in autumn, when the leafy growth was prevalent. In October 2018, the higher N, Mg, and Fe concentration may also be noted in the plant samples. The maximal quantum efficiency of PSII reached optimal values above 0.79 in all leaves including the control and treated plants, except a few cases, which could be due to environmental effects (e.g., drought) and not the treatments, as all treatment groups, including controls, were uniformly low (below 0.7). The improvement of the chlorophyll concentration and the functional state of the photosynthetic apparatus by sewage sludge applications have also been reported in previous studies [27,28].

MDA is a byproduct of lipid peroxidation, reflecting the failure of the cellular antioxidative apparatus to prevent oxidative stress [29]. MDA concentration significantly increased due to sewage sludge treatments in some sampling times. This may be attributed to high nutrient stress, as the concentration of toxic and trace elements was below the detection limit in the plant material. Micronutrients such as Mn, Cu, and Fe in May and July 2017 in Experiment 1 showed an increase in the plant biomass, partly simultaneous with that of MDA compared to the control, but in September, these changed in the opposite way. In Experiment 2, increased MDA levels were coupled with decreased concentrations of these elements. However, Na appeared to be increasing in parallel with MDA levels in all these sampling times. Na is not essential, but some plants adapted to increased concentrations in the soil to varying extent [30]. Szarvasi-1 plants showed considerable tolerance to NaCl treatments in a previous study, in which MDA levels did not increase up to 200 mM NaCl in the nutrient solution [16]. Nevertheless, the increase in MDA levels may reflect a cumulative effect of increased uptake and accumulation of macronutrients and Na.

### 3.2. Element Composition of Szarvasi-1 and the Sustainability of Sewage Sludge Applications

In the sewage sludge treatments, a significant nutrient transfer was made to the soil, with the assumption that a major portion will be recycled in plant biomass every year. We made an attempt to follow this nutrient cycling by sampling each container and each quadrate at five points, both at three soil depths, but indeed the total and soluble element concentrations did not show clear changes (Tables S1 and S2). There were considerable fluctuations in the untreated control containers and quadrates as well, which may be due to the effect of Szarvasi-1 plants mobilizing some of the elements and that the nutrient and trace elements partly remained bound to organic matter in the sludge particles. Previous studies have shown that sewage sludge application increases soil total and bioavailable nutrient content [25,31]. The increased yield data clearly show the positive effect of nutrients, which was coupled with the increase in the concentration of Ca, Mg, K, N, and S macronutrients in the harvested biomass. Interestingly, P did not increase (Experiment 1), and rather decreased (Experiment 2), based on the total average (Table 2 and Table 3), even though its concentration was comparable to N in the sewage sludge. This observation on the macroelements can also be observed at each sampling time. Nevertheless, in the first year of Experiment 2, Ca and Mg showed rather slight decrease compared to the control in the total aboveground biomass samples, suggesting that young plants do not require high concentrations of these elements along with P. But in the following years, as the plants became larger with a more efficient root system, more and more Ca and Mg were taken up, ending with a marked increase compared to the control, especially in the S2 and S3 treatments. This transition was not observed in Experiment 1, which might be explained by the smaller portions of sewage sludge being applied at different times.

K and Na seemed to behave as complementors. In most plants, K is preferred to Na, which is needed for the maintenance of cellular homeostasis, proper functioning of enzymes, and osmotic potential [30,32]. Szarvasi-1 energy grass is quite efficient in preserving a high K to Na ratio, even when Na is present in increased concentrations [16]. Nevertheless, as it efficiently tolerates Na, it might be accumulated and used for the osmotic balancing. It should also be noted that the majority of K accumulated in the tissues does not originate from the sewage sludge, which is proved by the CEUE calculation. As this ratio is between 2.5 and 8.2 for K across both experiments, the plants should have utilized the originally existing K pools in the soil. Indeed, K concentration was very low in the sewage sludge used in the present study compared to other macroelements. It is also possible that the elements showing decreases in the tissues, unparallel with others, were present in the sludge in non-readily plant-available form or became (at least temporarily) unavailable in the soil [33].

The microelement concentrations showed great variability in the tissue samples. It might be explained by the different amount of tissue types (stem, leaf, flower) in the total aboveground biomass samples due to seasonal variability. In Experiment 1, the samples of the summer harvest (July 2017) had increased concentrations for all microelements compared to the control (except for B due to missing data), whereas the following September brought a general decrease. In Experiment 2, the microelement concentrations showed increases only in later harvest occasions. Zn and Cu increased in most of the samples compared to the control in both experiments. The uptake and accumulation of microelements are strictly regulated in plants, as they are required in biochemical reactions and as components of enzymes, especially the redox-active ones, Fe, Mn, and Cu. The accumulation of these, as well as Zn, in tissues and cells may lead to oxidative stress unless they properly compartmentalized in the vacuoles or in the cell wall [34,35]. Previous studies showed that Szarvasi-1 plants are stimulated by high Zn concentrations in the nutrient solution, but Cu proved to be toxic when its availability is high [15,36]. However, there is no information on B, Mn, and Mo, nor on the tissue localization of the microelements accumulated at a supraoptimal level. Ni and Al generally decreased in the tissues compared to the control (except for July 2017 in Experiment 1, when both metals increased). The uptake and accumulation of, especially, the metal ions are dependent on their adsorption on negatively charged particles in the soil or in the root cell walls, and their bioavailability is also influenced by other factors in the soil mentioned previously [37]. Furthermore, when applied together, trace elements may influence the uptake and translocation of each other, which was already shown in Szarvasi-1 energy grass [17].

The balance between the amount of micro- and trace elements deposited on the soil with treatments and removed in the biomass was generally negative and decreased with increasing sewage sludge doses in both experiments. Exceptions are Fe and Ni. Fe showed more uniformity, meaning the removal rate changed very little, while Ni removal increased in some cases, especially in Experiment 1. Nevertheless, the CEUE was very low for all micro- and trace elements.

The surplus of nutrients not taken up by the plants remains in the soil and may undergo chemical changes, e.g., redox reactions depending on the soil redox potential or microbial activity, adsorption influenced by soil C.E.C., and may also be leached by precipitation, ultimately leading to eutrophication [38,39]. For this reason, the maximization of sewage sludge treatment doses should follow an optimal balance between input and removal by the energy plantation. Thus, the sustainability assessment of sewage sludge driven energy plantations relies on the plant side. More established conclusions require the investigation of soil processes and relevance of other risk factors such as microplastics and organic contaminants in the sludge affecting soil microbiota and even longer-term studies than the present one.

## 4. Materials and Methods

### 4.1. Soil, Plant Material, and Experimental Design

**Figure 7:**
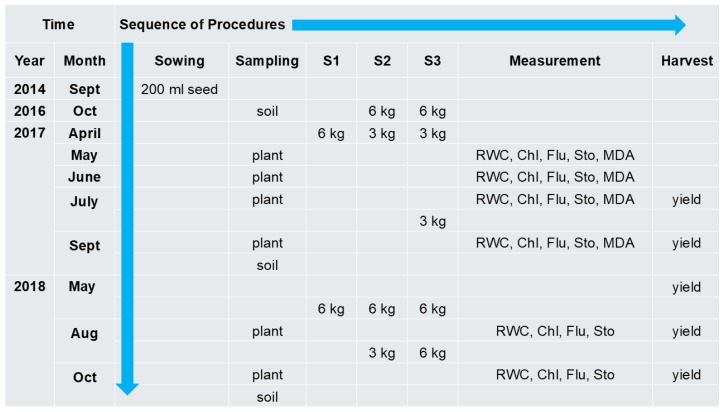
The timeline of the procedures made in Experiment 1. The sowing and treatments (S1–3) were applied to 1 m^3^ containers. RWC, relative water content; Chl, chlorophyll content (SPAD); Flu, chlorophyll a fluorescence induction; Sto, stomatal conductance; MDA, malondialdehyde.

All experiments were conducted outdoors. Experiment 1 was conducted in Szarvas, Hungary (GPS 46.828493 20.527978). This experiment aimed to address already developed plants and their response to high yearly dose of sewage sludge applied in several smaller portions. Aluminum containers without draining holes, to prevent leakage or interaction with local soil (V = 1 m^3^), were dug into the soil (only the upper 15 cm was above soil surface) and filled with homogenized soil. Soil samples were taken and analyzed for basic parameters in an international accredited analytical laboratory using Hungarian patents (Table S1). Element content in the soil, before the sewage sludge treatments and each year after the last harvest, was also determined in the accredited laboratory of the Budapest Sewerage Works PLC (Table S1). Szarvasi-1 energy grass seeds (200 mL ≈ 60 g) were sown on the soil surface two years before the commencement of the experiments. The plantations were exposed to actual weather conditions but were irrigated with tap water when rainfall was scarce. Soil samples (500 g) were taken from 0 to 20 cm, 20–40 cm, and 40–60 cm depth, and 3 containers of the same treatment were averaged to formulate 1 sample for each treatment. The treatments with dehydrated municipal sewage sludge derived from the Budapest Sewerage Works PLC were made uniformly distributed on the surface of the soil after the grass was cut (Table 1). Final treatment doses per year were 6 kg/9 kg/12 kg, denoted as S1/S2/S3 during 2016–2017, and it was repeated in 2018. The applied treatments are equivalent to 15, 22.25, and 30 Mg ha^−1^ dry sludge material. Untreated control containers are denoted as S0. The containers were watered periodically with tap water from spring to autumn, and in rainy weather, they were covered with a transparent roof (moved on rails). The harvest was performed with a handheld lawnmower, and the fresh biomass yield was weighed in each container. The timeline of the procedures including sowing, sampling, treatment, measurement, and harvest events is shown in (Figure 7).

**Figure 8:**
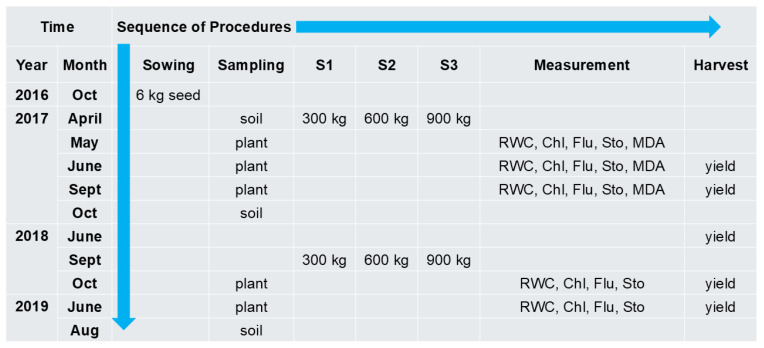
The timeline of the procedures made in Experiment 2. The sowing and treatments (S1–3) were made to 1 m^3^ containers. RWC, relative water content; Chl, chlorophyll content (SPAD); Flu, chlorophyll *a* fluorescence induction; Sto, stomatal conductance; MDA, malondialdehyde.

Experiment 2 was conducted in Budapest (GPS 47.573609 19.072769). This experiment addressed the response of young, developing plants to sewage sludge treatments, applied once a year within the range currently permitted for agricultural land under Hungarian regulations. Quadrates measuring 10 × 10 m were marked, and the soil was disced and then homogenized. Seeds of Szarvasi-1 were sown 1–2 cm below the surface of the soil with a seed drill (6 kg seed/quadrate) in October 2016. Soil samples (500 g) were taken from 5 points at 0–20 cm, 20–40 cm, and 40–60 cm depths and averaged at each quadrate to formulate one sample and then analyzed for basic parameters and element content before the sewage sludge treatments in 2016 and after the last harvest in 2017 and 2019, as in Experiment 1 (Table S2). Sewage sludge (same as in Exp. 1) treatments were also applied directly to soil surface dispersed homogeneously in the marked quadrates (Table 1). The applied doses were 300 kg/600 kg/900 kg, denoted as S1/S2/S3, in both 2017 and 2018. (The applied treatments are equivalent to 7.5, 15, and 22.25 Mg ha^−1^ dry sludge material). Untreated control quadrate was denoted as S0. The harvest was performed with a handheld lawnmower on 3 randomly selected areas of 1 m^2^ in each quadrate, and the fresh biomass yield was weighed immediately. The timeline of the procedures, including sowing, sampling, treatment, measurement, and harvest events, is shown in (Figure 8).

### 4.2. Relative Water Content and Yield Assessment

At each sampling, 3 parallel samples were taken per treatment (3 samples per quadrate, 1 sample per container). One sample contained all Szarvasi-1 aboveground plant material in a 10 × 10 cm sample area. Fresh weight was immediately recorded, while dry weight was measured after drying at 80 °C until constant weight was reached. The relative water content of the biomass was expressed as g H_2_O g^−1^ dry weight. Dry biomass yield was calculated on the basis of the dry matter content of the samples.

### 4.3. Chlorophyll Content

Relative chlorophyll content was measured with an SPAD chlorophyll meter (Konika-Minolta SPAD 502+; Tokyo, Japan) on the middle sections of the youngest fully developed leaves.

### 4.4. Stomatal Conductance

Stomatal conductance was measured with an AP4 porometer (Delta-T Devices, Cambridge, UK) on the adaxial epidermis of the middle sections of the youngest fully developed leaves. Conductance was calculated as mmol H_2_O m^−2^ s^−1^.

### 4.5. Malondialdehyde Concentration

MDA is accumulated due to the lipid peroxidation of polyunsaturated fatty acids, which is a commonly accepted marker of oxidative stress [40]. In this study, MDA concentration was quantified using a spectrophotometer and the reagent thiobarbituric acid (4,6-dihydroxi-2-merkaptopyrimidine) [41,42]. In total, 100 mg of leaf tissues, collected from the middle sections of the youngest fully developed leaves, were used, and the procedures to develop the reaction were performed as in Kolberg et al. [36]. The absorbance was measured at 532 nm with a Shimadzu UV-2101PC UV-VIS scanning spectrophotometer (Shimadzu, Kyoto, Japan). MDA content was determined based on the extinction coefficient ε = 155 mM^−1^ cm^−1^.

### 4.6. Chlorophyll a Fluorescence Induction

Chl *a* fluorescence induction on leaf samples were performed using an FMM Chl *a* fluorometer (Budapest University of Technology and Economics, Department of Atomic Physics, Budapest, Hungary) [43]. In order to determine the commonly used stress index Fv/Fm, i.e., the maximal quantum efficiency of PSII, we have applied the procedures and calculations described previously [36].

### 4.7. Measurement of Element Concentrations and Calculations

The concentration of elements in the sewage sludge was determined in the accredited laboratory of the Budapest Sewerage Works PLC as a standard protocol.

The concentration of elements in the soil and plant samples was determined with an inductively coupled plasma–optical emission spectrometer (ICP-OES) Thermo Scientific iCAP 7400 Thermoelemental (Thermo Fisher Scientific, Cambridge, UK). For the analytical preparations, Hungarian patents were used. Soluble fraction of the elements in the soil was determined in a solution extracted from the soil with deionized water (soil:H_2_O = 1:10 m/m%). Plant material was digested in the mixture of cc. HNO_3_ and cc. H_2_O_2_ (4:1) in a Mars 5 microwave digestion system (CEM, Matthews, NC, USA). A multielement standard was used for calibration (multi-element standard solution IV, Merck Reference material, Darmstadt, Germany) of 23 elements. For phosphorus, a total phosphorus standard (Merck Reference material) was used.

Cumulative element-use efficiency (CEUE), i.e., removal ratio, was calculated for 1, 2, or 3 years as follows:

$$\frac{\sum M_{i}\cdot c_{i}}{\sum M_{ss}\cdot c_{ss}}$$

where *M_i_* is the total aboveground biomass per unit area at harvest, *c_i_* is the concentration of an element in the biomass, *M_ss_* is the total amount of sewage sludge deposited on the unit area, and *c_ss_* is the concentration of the element in the sewage sludge.

### 4.8. Statistical Treatment

The experiments were conducted with 3 parallels: three identically treated containers in Experiment 1 and three independent samples from the 100 m^2^ quadrates of Experiment 2. Sample numbers for the instrumental measurements are indicated in the figure captions. In some cases, such as soil sampling, the independent samples were pooled to constitute one average sample per treatment and sampling occasion. Where applicable, statistical analysis was performed using the software package R (v2021.09.0). For the comparison of the data, the analysis of normality was performed using the Shapiro–Wilk test (at 99% level of confidence) and followed by Levene’s test for the homogeneity of variance (95% confidence level). After the verification, the data were evaluated by analysis of variance (ANOVA) followed by Tukey’s post hoc test. Statistically different groups (*p* < 0.05) are indicated by lowercase letters.

## 5. Conclusions

In the present work, two outdoor experiments were conducted to assess the efficiency of municipal sewage sludge in the sustainable fertilization of Szarvasi-1 energy grass plantations and the response of plants to the treatments. The plants physiologically tolerated well the high nutrient deposition in the containers and quadrates, and significant yield increment was reached in both setups. Based on the experiments, moderate doses of sewage sludge application, considering the element concentrations and balance in the sludge and soil, proved to be advantageous in new plantations. The concentration of macroelements Ca, Mg, N, and S increased in the aboveground biomass with increasing doses of sewage sludge, but even in three years, the amount removed with harvest occasions was much smaller than those remaining in the soil. Thus, most elements tended to accumulate in the soil, except for K, which was present in lower concentration in the sewage sludge than required by the plants, suggesting that extra K addition may be necessary when this sludge is used for soil fertilization. Micro- and trace-element concentrations did not show an increasing tendency in the biomass, implying a slower uptake and removal rate than those of macroelements. The results suggest that high-dose sewage sludge treatments in the Szarvasi-1 plantations may lead to the accumulation of nutrients and trace elements in the soil prone to leaching, and instead, yearly treatments in lower doses in several-year-old existing plantations may be a more viable solution. It should be noted that in this study no significant element accumulation was evidenced in the soil, and the increase in element content was indirectly concluded from the balance of input and output.

## Figures and Tables

**Table 1:** Concentration of elements, organic contaminants, and the pH in the sewage sludge applied in the treatments. The maximum allowed concentration in the sewage sludge (L1, limit of concentration [mg kg^−1^ d.m.]) and the maximum allowed dose of deposited elements (L2, limit of application [kg ha^−1^ year^−1^]) are shown as reference values [7].

Dry matter content	[kg d.m. kg^−1^]	0.255 ± 0.014		
pH		8.58 ± 0.22		
**Element**		**Concentration**	**L1**	**L2**
Ca	[g kg^−1^ d.m.]	55.55 ± 1.34		
Mg	[g kg^−1^ d.m.]	7.26 ± 0.01		
K	[g kg^−1^ d.m.]	2.43 ± 0.85		
N	[g kg^−1^ d.m.]	45.25 ± 2.85		
P	[g kg^−1^ d.m.]	30.79 ± 8.55		
S	[g kg^−1^ d.m.]	15.15 ± 1.20		
Na	[g kg^−1^ d.m.]	5.26 ± 0.00		
B	[mg kg^−1^ d.m.]	29.95 ± 5.43		
Mn	[mg kg^−1^ d.m.]	209.25 ± 34.83		
Zn	[mg kg^−1^ d.m.]	669.00 ± 164.97	2500	30
Cu	[mg kg^−1^ d.m.]	371.75 ± 48.22	1000	10
Fe	[mg kg^−1^ d.m.]	3520.00 ± 890.95		
Ni	[mg kg^−1^ d.m.]	30.60 ± 7.52	200	2.0
Al	[mg kg^−1^ d.m.]	4143.25 ± 2313.96		
Mo	[mg kg^−1^ d.m.]	5.18 ± 1.07	20	0.2
As	[mg kg^−1^ d.m.]	5.52 ± 1.08	75	0.5
Cd	[mg kg^−1^ d.m.]	2.09 ± 0.69	10	0.15
Co	[mg kg^−1^ d.m.]	5.17 ± 3.23	50	0.5
Cr	[mg kg^−1^ d.m.]	33.15 ± 10.48	1000	10
Hg	[mg kg^−1^ d.m.]	0.71 ± 0.06	10	0.1
Pb	[mg kg^−1^ d.m.]	44.60 ± 2.31	750	10
**Organic contaminant**				
TPH ^1^	[mg kg^−1^ d.m.]	3890.00 ± 169.71	4000	40
PAH ^2^	[mg kg^−1^ d.m.]	1.82 ± 0.38	10,000	0.1
PCB ^3^	[mg kg^−1^ d.m.]	<0.01	1	0.05

^1^ Total Petroleum Hydrocarbons; ^2^ Polycyclic Aromatic Hydrocarbon; ^3^ Polychlorinated Biphenyls.

**Table 2:** Element concentrations (mg kg^−1^) in the aboveground biomass of Szarvasi-1 energy grass treated with increasing doses of sewage sludge (S1–3) in Experiment 1. Data are presented as mean ± SD of the concentrations measured in the samples at all harvest occasions (n = 6). There are no significant differences between the treatments at *p* < 0.05 (one-way ANOVA with Tukey’s HSD post hoc test).

Element	Treatments			
	S0	S1	S2	S3
Ca	6202 ± 1223	7646 ± 1363	8010 ± 1429	8121 ± 2210
Mg	1453 ± 358	1907 ± 519	2055 ± 544	2020 ± 453
K	24,967 ± 5643	31,886 ± 7640	29,420 ± 9933	32,776 ± 9506
N	19,437 ± 5532	29,405 ± 8970	29,570 ± 9972	28,824 ± 8885
P	2334 ± 316	2154 ± 577	2140 ± 497	1982 ± 386
S	2338 ± 705	3195 ± 656	4488 ± 2226	2975 ± 1051
Na	2874 ± 601	4103 ± 2521	6441 ± 2302	5345 ± 2702
B	25.09 ± 18.38	33.97 ± 33.43	28.52 ± 26.07	26.92 ± 20.05
Mn	23.48 ± 12.20	22.03 ± 5.77	24.63 ± 11.10	23.89 ± 10.57
Zn	18.96 ± 5.49	21.25 ± 6.98	24.79 ± 10.95	27.09 ± 12.70
Cu	8.29 ± 4.47	10.00 ± 6.36	10.69 ± 6.58	11.05 ± 7.26
Fe	96.32 ± 54.42	122.91 ± 59.13	139.52 ± 79.01	131.20 ± 65.91
Ni	2.12 ± 1.97	0.96 ± 0.55	1.19 ± 0.66	1.74 ± 1.88
Al	68.90 ± 37.97	74.53 ± 44.74	68.84 ± 41.10	78.77 ± 50.11
As	1.22 ± 0.32	1.28 ± 0.63	1.27 ± 0.61	1.22 ± 0.48
Cd	0.22 ± 0.05	0.21 ± 0.01	0.21 ± 0.02	0.20 ± 0.00
Pb	2.25 ± 0.75	1.87 ± 0.27	1.60 ± 0.40	2.59 ± 1.00

**Table 3:** Element concentrations (mg kg^−1^) in the aboveground biomass of Szarvasi-1 energy grass treated with increasing doses of sewage sludge (S1–3) in Experiment 2. Data are presented as mean ± SD of the concentrations measured in the samples at all harvest occasions (n = 4). There are no significant differences between the treatments at *p* < 0.05 (one-way ANOVA with Tukey’s HSD post hoc test).

Element	Treatments			
	S0	S1	S2	S3
Ca	7508 ± 3165	7851 ± 3056	8354 ± 2551	9224 ± 3962
Mg	1468 ± 640	1363 ± 540	1770 ± 924	1917 ± 981
K	22,285 ± 7134	24,001 ± 8272	21,917 ± 8707	23,853 ± 8407
N	17,850 ± 12,857	25,943 ± 13,023	28,170 ± 14,867	31,390 ± 16,206
P	2514 ± 497	2131 ± 189	2044 ± 445	1863 ± 370
S	3115 ± 771	3225 ± 1181	3505 ± 1747	4755 ± 431
Na	1167 ± 715	857 ± 503	1940 ± 201	2687 ± 1880
B	16.32 ± 10.61	20.58 ± 17.97	15.26 ± 7.13	15.16 ± 11.38
Mn	20.48 ± 13.74	10.76 ± 3.76	21.24 ± 16.13	19.98 ± 16.10
Zn	25.12 ± 12.25	26.89 ± 11.86	34.54 ± 29.73	30.00 ± 11.87
Cu	5.92 ± 2.28	7.76 ± 2.64	8.77 ± 4.24	8.01 ± 2.45
Fe	59.92 ± 38.56	67.02 ± 40.34	60.78 ± 48.59	76.00 ± 58.02
Ni	1.33 ± 0.58	0.91 ± 0.91	1.06 ± 0.38	0.63 ± 0.23
Al	129.30 ± 123.26	113.01 ± 137.02	109.59 ± 120.50	123.27 ± 162.02
As	1.00 ± 0.00	1.74 ± 1.48	1.54 ± 1.08	1.60 ± 1.20
Cd	0.22 ± 0.04	0.26 ± 0.09	0.20 ± 0.00	0.22 ± 0.03
Pb	0.46 ± 0.51	0.45 ± 0.51	0.56 ± 0.68	0.48 ± 0.55

**Table 4:** Ratio of the total amount of elements introduced to the containers in Experiment 1 with sewage sludge treatments (S1–3) to that harvested in the aboveground biomass of Szarvasi-1 energy grass (CEUE) over one year (2017) and two years (2017–2018) of the experiments.

Element	Treatments					
		2017			2017–2018	
	S1	S2	S3	S1	S2	S3
Ca	0.065	0.056	0.046	0.060	0.053	0.044
Mg	0.127	0.115	0.093	0.123	0.111	0.086
K	7.247	5.367	4.939	6.250	5.178	4.420
N	0.285	0.265	0.196	0.298	0.269	0.195
P	0.049	0.042	0.031	0.035	0.033	0.023
S	0.102	0.193	0.061	0.089	0.142	0.053
Na	0.349	0.464	0.366	0.424	0.519	0.371
B	0.177	0.154	0.122	0.336	0.253	0.091
Mn	0.061	0.054	0.046	0.054	0.054	0.041
Zn	0.012	0.011	0.009	0.014	0.015	0.012
Cu	0.010	0.009	0.008	0.014	0.012	0.010
Fe	0.025	0.028	0.019	0.017	0.018	0.013
Ni	0.029	0.028	0.059	0.016	0.016	0.028
Al	0.007	0.007	0.069	0.100	0.088	0.061
As	0.101	0.088	0.069	0.100	0.088	0.061
Cd	0.088	0.076	0.060	0.051	0.044	0.034
Pb	0.024	0.015	0.016	0.016	0.010	0.012

**Table 5:** Ratio of the total amount of elements introduced to the quadrates in Experiment 2 with sewage sludge treatments (S1–3) to that harvested in the aboveground biomass of Szarvasi-1 energy grass (CEUE) over one year (2017), two years (2017–2018), and three years (2017–2019) of the experiments.

Element	Treatments								
	2017			2017–2018			2017–2019		
	S1	S2	S3	S1	S2	S3	S1	S2	S3
Ca	0.091	0.054	0.041	0.100	0.065	0.048	0.118	0.080	0.058
Mg	0.132	0.086	0.062	0.136	0.111	0.076	0.162	0.127	0.094
K	8.210	4.521	3.614	5.543	3.240	2.247	6.583	3.751	2.759
N	0.201	0.148	0.134	0.268	0.213	0.164	0.309	0.251	0.199
P	0.036	0.021	0.014	0.034	0.022	0.013	0.046	0.029	0.017
S	0.147	0.093	0.073	0.297	0.205	0.165	0.408	0.287	0.253
Na	0.059	0.127	0.074	0.094	0.157	0.137	0.167	0.221	0.216
B	0.244	0.152	0.100	0.316	0.183	0.103	0.735	0.335	0.256
Mn	0.038	0.023	0.015	0.029	0.038	0.023	0.039	0.045	0.028
Zn	0.014	0.008	0.007	0.024	0.021	0.011	0.031	0.024	0.015
Cu	0.009	0.006	0.004	0.012	0.009	0.005	0.014	0.010	0.007
Fe	0.002	0.001	0.001	0.002	0.002	0.001	0.002	0.002	0.001
Ni	0.007	0.010	0.004	0.010	0.011	0.005	0.026	0.018	0.006
Al	0.003	0.002	0.001	0.016	0.012	0.007	0.044	0.028	0.024
As	0.079	0.045	0.032	0.192	0.107	0.071	0.234	0.135	0.093
Cd	0.064	0.036	0.028	0.065	0.031	0.022	0.085	0.045	0.033
Pb	0.013	0.009	0.005	0.009	0.006	0.003	0.010	0.007	0.004

## Data Availability

The data presented in this study are available on reasonable request from the corresponding author.

## Supplementary Materials

The following supporting information can be downloaded at https://www.mdpi.com/article/10.3390/plants15030392/s1. Table S1: Soil characteristics of the containers in Experiment 1 examined before the experiment, including total element concentrations and soluble element concentrations (H_2_O 1:10) at all sampling times. Table S2: Soil characteristics of the study quadrates in Experiment 2 examined before the experiment, including total element concentrations and soluble element concentrations (H_2_O 1:10) at all sampling times.
